# Could the kynurenine pathway be the key missing piece of Myalgic Encephalomyelitis/Chronic Fatigue Syndrome (ME/CFS) complex puzzle?

**DOI:** 10.1007/s00018-022-04380-5

**Published:** 2022-07-11

**Authors:** Bahar Kavyani, Brett A. Lidbury, Richard Schloeffel, Paul R. Fisher, Daniel Missailidis, Sarah J. Annesley, Mona Dehhaghi, Benjamin Heng, Gilles J. Guillemin

**Affiliations:** 1 Neuroinflammation Group, Department of Biomedical Sciences, Macquarie University, Sydney, Australia; 2 The National Centre for Epidemiology and Population Health, RSPH, College of Health and Medicine, The Australian National University, Canberra, ACT 2601 Australia; 3 The Grove Health Pymble, Sydney, NSW Australia; 4 Department of Physiology, Anatomy and Microbiology, La Trobe University, Melbourne, Australia; 5 Pandis.org, Melbourne, Australia

**Keywords:** Myalgic Encephalomyelitis/Chronic Fatigue Syndrome, Kynurenine pathway, Neuroinflammation, Mitochondrial dysfunction, Immune dysregulation, Tryptophan

## Abstract

Myalgic Encephalomyelitis/Chronic Fatigue Syndrome (ME/CFS) is a complex and debilitating disease with a substantial social and economic impact on individuals and their community. Despite its importance and deteriorating impact, progresses in diagnosis and treatment of ME/CFS is limited. This is due to the unclear pathophysiology of the disease and consequently lack of prognostic biomarkers. To investigate pathophysiology of ME/CFS, several potential pathologic hallmarks have been investigated; however, these studies have failed to report a consistent result. These failures in introducing the underlying reason for ME/CFS have stimulated considering other possible contributing mechanisms such as tryptophan (TRP) metabolism and in particular kynurenine pathway (KP). KP plays a central role in cellular energy production through the production of nicotinamide adenine dinucleotide (NADH). In addition, this pathway has been shown to mediate immune response and neuroinflammation through its metabolites. This review, we will discuss the pathology and management of ME/CFS and provide evidence pertaining KP abnormalities and symptoms that are classic characteristics of ME/CFS. Targeting the KP regulation may provide innovative approaches to the management of ME/CFS.

## Myalgic Encephalomyelitis/Chronic Fatigue Syndrome (ME/CFS)

ME/CFS is a complex and debilitating disease that has a substantial social and economic impact on individuals and their community. In Australia, the estimated burden of the disease cost is about $14.5 billion annually in lost income and medical bills (approximately 70% and 30% of the total cost, respectively [1]. Patients with ME/CFS usually presented with symptoms such as lowered capacity for daily activity along with fatigue, unrefreshing sleep, post-exertional malaise (PEM), and cognitive dysfunction lasting for more than 6 months [1]. This disease tends to affect both children and adults, with two age peaks ranged from 10 to 19 years and 30–39 years [2]. The relative risk for females suffering from ME or CFS compared to males is reported between 1.238 (95% CI 1.235–1.242) and 3.2 (95% CI 3.0–3.4) in different studies [2, 3].

The prevalence of the disease differs greatly between countries ranging from 0.03 to 6.41%. In one recent study conducted in 2019, an estimated prevalence has been reported 1% of the population worldwide [3]. In Australia, this number has been estimated to be at 0.76%, which means approximately 191,544 Australians are living with ME/CFS [1]. The discrepancy in case numbers can be attributed to a lack of consensus in case definition and criteria, the complexity and the unknown cause of the disease. This review aims to provide an overview of the current understanding of the pathology and treatment of ME/CFS. Given that the underlying mechanism(s) of the disease pathology remains inconclusive, this review will highlight an alternative mechanism, the kynurenine pathway (KP), and discuss its potential roles in the pathology of ME/CFS. We will discuss the correlation between abnormal levels of KP metabolites and symptoms that are hallmarks of ME/CFS.

## Diagnostic criteria for ME/CFS

At present, there are 25 established diagnostic criteria developed based on the disease’s clinical features. The criteria can be classified into four groups: ME, CFS, ME/CFS, and systemic exertion intolerance disorder (SEID). According to clinical characteristics, ME is associated with infection; ME/CFS with neuroinflammatory disorders; CFS with unknown causes, and SEID with fatigue or malaise [4]. The term ME/CFS is being used by Emerge Australia to include both conditions with possible subgroups. Emerge Australia is the national patient organization for ME/CFS, raising awareness, as well as providing financial and social support for the patients. Emerge Australia advocates the use of the US National Academy of Medicine (NAM) criteria for clinical use, and the Canadian Consensus Criteria (CCC) for research, in which PEM has been highlighted as the defining feature of the disease. According to NAM criteria, the minimum clinical criteria to be diagnosed with ME/CFS are: (1) PEM, (2) a substantial reduction or impairment in the ability to engage in pre-illness levels of occupational, educational, social, or personal activities that persist for more than 6 months and is accompanied by fatigue, which is often profound, is of new onset (not lifelong), is not the result of ongoing excessive exertion, and is not substantially alleviated by rest (3) unrefreshing sleep (4) at least one of the cognitive impairment or orthostatic intolerance. Although the diagnosis criteria have been established, not all of them have been subjected to systematic evaluation [5]. In addition, this criterion is based on the symptoms but not the cause, and based on what patients report in their questionnaires. The cause of the disease has remained unclear, as the reports on the pathology characteristics are controversial or were based on insufficient research setup/sample size. This will be discussed below.

## Current treatments

Due to the lack of known causes, diagnostic biomarkers, and absence of consensus on case definitions, there is no approved treatment for ME/CFS. In addition, various treatments being used currently including immunologic, pharmacologic, behavioural treatments as well as complementary and alternative medicine, are off-label (without approved indication in ME/CFS). Therefore, lifestyle changes (such as sleep hygiene, psychotherapies, socialising, balanced diet, nutritional consideration, exercise) and patient education regarding the disease, are recommended as the first initiatives by physicians [6]. ME/CFS treatments mainly target symptoms (including fatigue, sleep disorders, pain, cognitive disorders, immune dysfunction), while others aim for the cause of ME/CFS, as reported by FDA reports in 2015.

### Symptom-based therapy

Pharmaceutical treatments prescribed are usually based on symptoms that includes sleep disorders, pain, fatigue, and cognitive disorders. The prescribed treatments are: sedatives, anti-depressants, sedative-hypnotics for sleep disorders; analgesics, anti-depressants, NSAIDs (Non-Steroidal Anti-Inflammatory Drugs) and anticonvulsant for pain; stimulants and vitamin/mineral supplements for fatigue, and finally stimulants and calcium channel blockers are for cognitive disorders [7]. In addition to pharmacological interventions, many non-pharmacological therapies have been studied; including psychological methods (i.e., Cognitive behaviour therapy (CBT)) and exercise (such as Graded exercise therapy (GET), rehabilitation and acupuncture) [8, 9]. However, none of the proposed therapies are of proven benefit to all patients and some, particularly GET, may worsen symptoms and illness progression by causing post-exertional malaise [10]. Most importantly there is now clear and increasing evidence that molecular cytopathologies play key roles in the underlying disease mechanisms in ME/CFS [11, 12]. Pharmaceutical and non-pharmaceutical interventions which are predominantly prescribed to alleviate various symptoms are introduced in Table1.

**Table 1:** Current treatments available to treat symptoms in ME/CFS patients

Symptoms	Sleep disorders	Pain	Fatigue	Cognitive disorders
Pharmaceutical interventions	Amitriptyline, zolpiclone, trazodone, doxepine, clonazepam, Cyclobenzaprine, l-Tryptophan	Panadol, nortriptyline, doxepin, amitriptyline, NSAIDs, gabapentin, cyclobenzaprine	Methylphenidate, modafinil, cyano-cobalamin, dextro-amphetamine,	Methylphenidate, Modafinil, detro-amphetamine, nimodipine
Non-pharmaceutical interventions	Addressing sleep problems (such as sleep apnea), sleep hygiene, psychotherapies, acupuncture, meditation	massage Therapy, physiotherapy, chiropractic adjustments, relaxation techniques and meditation, Synaptic Electronic Activation Technology (SEA Tech.) The magnetic pulser MPG4, Bio-Resonance therapy	Sleep management, psychotherapies, socialising, balanced diet, nutritional consideration, exercise (GET), acupuncture	Techniques regarding mental exercise such as reading or learning, mediation, CBT

### Treating the potential cause

The pharmaceutical treatments that target the potential cause of ME/CFS comprise the use of antivirals and antibiotics medications. Several viruses have been reported in ME/CFS patients as the triggering infections contributing to a subgroup of ME/CFS patients. These viruses are mainly herpes simplex virus (i.e. human cytomegalovirus (CMV) and Epstein–Barr virus (EBV)), enteroviruses, human parvovirus B19, Ross River virus [13]. Therefore, antivirals such as Essential Fatty Acids (EFA), and ganciclovir have been used to eliminate viral infections. EFA application was studied in two human trial studies using high doses of evening prim-rose oil containing γ-linolenic acid (GLA), and fish oil concentrate versus placebo [14, 15]. Results of these studies were contradicting regarding overall improvements in symptoms (fatigue, myalgia, dizziness, poor concentration, and depression). First study in 1990 reported significant improvement (P < 0.0001), while the second trial in 1991 showed no significant differences between treated and placebo groups. This contrasting data could be due to the different case definition criteria and formulation of placebo (paraffin in the first study, sunflower oil in the second trial) utilized [16]. On the contrary, improvements in fatigue and cognitive symptoms with valganciclovir have been reported in several studies. For example, in a randomized trial of 30 CFS patients, significant differences between groups were observed in Multidimensional Fatigue Inventory (MFI-20)(P = 0.039), Fatigue Severity Scale (FSS) scores (P = 0.006), and cognitive function (*p* = 0.025) [17]. However, these studies did not achieve significant observations between treatment and placebo due to the small sample size, additional studies are required to confirm the results associated with valganciclovir [17–19].

Various bacterial infections involved in ME/CFS have been reported. In 2002, Garth Nicolson and co-workers reported a High prevalence of Mycoplasma infections in ME/CFS patients [20]. In 2007, they introduced Borrelia burgdorferi and Mycoplasma species as the dominant infections in subgroups of ME/CFS patients [21]. Similarly, they reported Mycoplasma and Chlamydia infections in addition to viral infections in these patients as the common infections in another study [22]. Depending on the initial infection, different antibiotics are prescribed in these patients, targeting chronic infections and chronic symptoms of fatigue. The antibiotics commonly used are doxycycline (100 mg bid-tid), ciprofloxacin (750 mg bid), clarithromycin (750–100 mg daily on a q12h schedule), and azithromycin (500 mg as a single dose) in treatment of the confirmed infections. Usually, the antibiotic regimen prescribed for patients are for 6 months or until improvement is noted, then repeated periods of 6 weeks with 2 weeks intervals [6, 23]. In one study, for instance, out of 171 patients suffering from Chlamydia pneumoniae ten patients who showed symptoms of ME/CFS were treated with azithromycin therapy. And all showed favourable clinical and serological responses to treatment [24]. However, none of these infections has been verified as the main cause, and more investigations in pathology of ME/CFS is needed.

In addition, many genetic disorders can cause various symptoms in ME/CFS patients. Some evidence indicated that Celiac disease, as a genetically inherited intolerance to Gluten, can generate neurological symptoms in the absence of GI symptoms in these patients [25]. Moreover, Mitochondrial neurogastrointestinal encephalopathy (MNGIE) can be responsible for GI disturbance and it has been reported to be present in a subgroup of ME/CFS patients. In addition, single nucleotide polymorphisms (SNPs), or heteroplasmy of mitochondrial DNA (mtDNA) may lead to the exhibition of certain neurological, inflammatory, and/or GI symptoms [26]. However, none of them has been approved as the main cause, and various treatments have been suggested according to the manifestation of each disorder in these patients.

### Treatments under investigation

Other candidate pharmacologic interventions have been studied in placebo-controlled trials and they are rintatolimod, rituximab, and various nutritional supplements. Among the above drugs, rintatolimod (Ampligen) showed the most promising improvements and benefits in treating different symptoms of ME/CFS. Rintatolimod is a nucleic acid (double-stranded RNA) compound which acts as an inducer of interferon. Mechanism of action of rintatolimod is enhancing the NK-cell function and antiviral effects via influencing the 2-5A-synthetase [27]. Clinical trials reported that patients treated with rintatolimod showed improvement in fatigue (measured by exercise tolerance test) and quality of life in a subset of patients with CFS/ME, as well as postponing progression of the disease (NCT00215813 and NCT00215800). However, its administration alone has not been approved as more efficacy and safety data have been requested by the FDA [27–30]. This could be due to the small number of patients involved in these studies and the possibility of outweighing the side effects over the benefits, success in finding treatment options was minimal [31]. There have also been suggestions to combine this drug into regimens due to multi-factorial pathogenic basis of the disease [32].

Another important immune modulator which has been studied in randomized clinical trials (RCTs) is rituximab. Rituximab as a monoclonal antibody is designed to reduce inflammation via bonding to CD20 receptors on B-cells and their depletion. It was initially administrated for non-Hodgkin’s lymphoma and autoimmune disorders [33]. Accidently, it was used for the first time for a patient suffering from both lymphoma and ME/CFS by Dr Fluge. Interestingly, this patient showed improvement in his fatigue. Thereafter, Fluge et al*.,* conducted three studies in 1997, 2009, and 2015 to investigate the effects of rituximab in ME/CFS patients. They reported overall symptoms improvement in 67% of patients in their largest study including 30 patients [34–36]. Despite the benefits related to rituximab, its safety profile is a concern regarding suppressing immune response and increasing the chance of infection, as well as neutropenia reported as its side effect previously. Recently, a multicentre study in Norway recruited 151 patients to extend the study on the safety of Rituximab in a larger population. There was no significant improvement between the two arms of the study (rituximab vs. placebo). In addition, 26.0% of the patients in the rituximab group and 18.9% of patients in the placebo group showed serious side effects [37]. As opposed to these medications with a high risk of side effects occurrence, nutritional supplements offer a much safer profile.

#### Nutritional supplements

Given that many nutrient deficiencies [such as vitamin C, vitamin B complex, sodium, magnesium, zinc, folic acid, l-carnitine, l-tryptophan (TRP), EFA, and coenzyme Q10 (CoQ10)] have been reported in patients with ME/CFS, several nutritional components have been studied in RCTs to alleviate symptoms in ME/CFS patients [38]. One of the examined nutritional supplements for treatment in ME/CFS is the Acetyl-l-carnitine (ALC). It is a transporter of fatty acid and is involved in the energy production by catabolising lipids in the brain and muscles. Some success was reported by various patients in reducing both mental and physical fatigue after treatment with ALC [39, 40]. In a study conducted in 2008, differences between ALC-treated and placebo-treated patients for the following parameters were: muscle pain − 27% versus − 3% (*p* = 0.02); prolonged fatigue after exercise: 51% versus − 4% (*p* < 0.001); sleep disorders: 28% versus 4% (*p* < 0.05); physical fatigue: 7 versus − 0.5 (*p* < 0.001); mental fatigue: − 3.3 versus 0.6 (*p* < 0.001); fatigue severity scale: − 22.5 versus 1.2 (*p* < 0.001); functional status 17.1 versus 0.6 (*p* < 0.001); MMSE 3.4 versus 0.5 (*p* < 0.001), which highlights the benefit of ALC treatment in improving both the physical and mental fatigue.

The other nutrition supplement that is examined in ME/CFS is the intramuscular injections of magnesium to relieve muscular symptoms [41]. Contrasting results were reported in a different subgroup of patients, as the magnesium deficiency played an important role in the efficacy of using magnesium supplements in alleviating the symptoms. Those patients who had magnesium deficiency benefited from the treatment, while others found it not useful [42–44].

Vitamin B12 has been proposed as an effective agent in reducing fatigue since the 1950s. It has been studied both as monotherapy or in combination with other components such as folic acid in multiple studies in ME/CFS [33]. These studies reported different results due to the usage of different active forms of vitamin B12, different combinations with various drugs, as well as various doses and routes of administration. Among them, two studies stand out: one study was 15 years’ experience of treating ME/CFS patients with vitamin B12 (methylcobalamin, as the biologically active form) in which patients reported less fatigue using the Fibro Fatigue (FF) scale after receiving these injections [45]. Another study conducted by the same group in 2015, administrated frequent injections of highly concentrated methylcobalamin combined with an individual daily high dose of oral folic acid, and reported the efficacy of this intervention in reducing fatigue in the patients. However, performing additional tests such as blood tests to adjust the dosage for each patient according to their deficiencies and other co-exciting conditions [45].

Treatment targeting oxidative stress is another approach to improve symptoms and the quality of life in patients with ME/CFS [46]. This includes the use of multivitamins and mineral supplements as antioxidants to boost mitochondrial function and lessen cellular damage by oxidative stress. One of the antioxidant agents that have gained a lot of attention is oral nicotinamide adenine dinucleotide (NADH) in ME/CFS patients. Effective symptoms relief was reported in a study conducted in 1999 in which patients received the treatment for 12 weeks [47]. Another study compared this treatment with other conventional supplements for 24 months in 31 patient with ME/CFS, and found it much more effective [48]. A shorter time frame study reported oral administration of 20 mg per day of NADH effective in reducing anxiety in ME/CFS patients [49]. However, a review paper in 2011 did not find enough evidence for the efficacy of this treatment due to shortcomings of these RCTs, as there were insufficient evidence and no data on functional status and quality of life to recommend these supplements as a treatment in chronic fatigue syndrome [50]. In addition, NADH in combination with CoQ10 was studied as the common antioxidants and booster of mitochondrial function. Consequently, various studies reported benefits associated with this regimen in patients [51–53]. For example, in an 8-week, randomized, controlled, a double-blind trial which was conducted on 80 CFS patients, those who were assigned to receive CoQ10 plus NADH supplementation (twice daily) showed a decrease in the perception of fatigue through all follow-up visits, when compared to the placebo group [53].

The nutraceutical was introduced as the combination of food supplements containing sodium salt of dichloroacetate (DCA) plus 100 mg Vitamin B1. Afterwards, this formulation was extended by adding alfa-lipoic acid (ALA), ALC and the oxidoreductase ubiquinone Q10, to boost mitochondrial function. Out of 22 patients, 10 patients experienced significant improvement by 50%, which highlighted the role of mitochondrial hypo-metabolism in the pathogenesis of primary ME/CFS [54]. However, these studies proposed that this treatment is not effective for all the patients, and it would be only effective in a specific subgroup of patients which were determined by a pre-determined formula [55]. In a similar study, symptoms were compared before and after treatment in 135 ME/CFS patients after receiving either KPAX002 (combination of methylphenidate and mitochondrial nutrients) or a placebo for 12 weeks. Change in the Checklist Individual Strength (CIS) and visual analogue scales for fatigue, as well as concentration disturbance symptoms, were investigated in these patients. As a result, reduction in fatigue and concentration symptoms were occurred after treatment [56]. Similarly, another study highlighted the importance of addressing mitochondrial dysfunction in 138 ME/CFS patients after taking ATP to profile biomedical test. These patients were prescribed a personalized regimen of various nutrients according to the type of their mitochondrial impairments (either co-factor deficiency or chemicals). All the patients who followed the treatment showed improvement in their mitochondrial energy score [57].

The main problem with studies investigating the efficacy of these supplements in ME/CFS is that the size of RCTs is not large enough. In addition, patients with different profiles, coexisting conditions and deficiencies responded differently to the treatments; therefore, therapeutic dosage should be adjusted in a personalized treatment.

One study suggested the combination of psychological and nutritional techniques for the treatment of ME/CFS, as it has reported improvements in fatigue, functional ability, and symptomatology. However, this study was not randomized and had no control group, therefore, requires proof from other randomized studies [58].

#### Microbiome supplements

The human microbiome is made up of a huge variety of microorganisms such as bacteria and archaeal. Microbiome has been shown to play a critical role in the balance of healthy and disease state by influencing directly or indirectly with the immune system, metabolic functions and protection against pathogens [59]. Given that one of the symptoms of ME/CFS is gut disorders, faecal microbiota transplantation (FMT) treatment was proposed due to reported improvements. FMT is the infusion of liquid filtrate faeces from a healthy donor into the gut of a recipient to target microbiota-gut-brain axis [31]. Two studies were conducted by Smith and co-workers, in which symptom improvement was reported after a time lapse of 15–20 years. 60 ME/CFS patients who were given FMT reported 50% and 70% significant symptom improvement in these two studies [60].

Alternative to FMT is immune stimulation therapy with the use of bacteria toxin such as the Staphylococcal toxoid vaccine. This vaccine generates a serological response to several staphylococcal antigens, particularly to certain extracellular toxins and enzymes. Although immune responses in ME/CFS patients have not been agreed on, responses to this vaccine have been reported to be fruitful in the clinical outcome of treatment. There were two RCTs that have examined its impact on a patient with ME/CFS. The first RCT reported significant benefits to the patients whereby improvement in pain and psychometric assessment was reported after vaccination when compared to controls receiving sterile water injections [61]. A second RCT study continues with the follow-up of the patients in the first RCT for 6 months after the administration of the second shot. The outcome of the second study shows a highly favourable outcome where 50% of patients were more functional by rehabilitating successfully and resuming half-time or full-time work confirmed the benefits for the patients [40].

#### Opioid antagonist treatment

Low doses of Naltrexone (LDN) are used as an off-label treatment for several chronic immune-modulated disorders including Crohn’s disease, fibromyalgia (FM), and Gulf War Illness. Therefore, it has been investigated in ME/CFS and a positive treatment response was reported with low dose naltrexone (LDN) (3.0–4.5 mg/day) in 73.9% of the patients (218 ME/CFS patients) regarding improvement in their vigilance/alertness, as well as improved physical and cognitive performance. Mild adverse effect and the high frequency of positive reports suggested LDN as a possible treatment for ME/CFS [62].

#### Surgical treatment

In severe cases of ME/CFS, surgical treatment of cervical spinal stenosis led to a marked improvement in myelopathic symptoms, resolution of light-headedness and hemodynamic dysfunction, improvement in activity levels and in global ME/CFS symptoms. This result introduced cervical spine stenosis as a contributing factor to the pathogenesis of refractory ME/CFS and orthostatic symptoms, which emphasizes the importance of myelopathy examination [63].

## Major pathological hallmarks of ME/CFS

### Dysfunctions of the immune system

It is well accepted that the immune system plays a key role in ME/CFS to either resolve the damage incurred during the development of the disease or in response to the disease trigger [12]. The immune cell population and cytokine levels have been studied in patients with ME/CFS to correlate the immunological data with the pathological characteristics of the disease. One of the major findings on immunological response is a shift from T helper type 1 (Th1) to Th2. Th1 and Th2 are subsets of T cells which are involved in the cellular immune response, in which Th1 is involved in the elimination of foreign bodies with IFN-γ as their primary cytokine, while Th2 is involved in atopy and anti-inflammatory response, producing interleukins (IL) 4, 5, 10 and 13 A case in point is a study reporting a skewed in T helper polarisation towards a Th2 type of immune response in 114 ME/CFS patients. Furthermore, patients experienced more severe symptoms, such as poor sleep [64]. Change was also observed in the CD8 + Mucosal Associated Invariant T cells (MAIT) cells, which is a type of innate-like T cells which have a protective role during microbial infection. However, the findings from the two different studies are inconclusive [65, 66].

Another major immune population involved in ME/CFS are B-cell lymphocytes. B-cell involvement was emphasised by two clinical trials (NCT01156909 and NCT00848692) that investigated B-cell depletion via monotherapy and maintenance using Rituximab, a monoclonal anti-CD20 antibody. Both trials reported significant treatment response with substantial improvement in ME/CFS symptoms [34, 67]. This result suggested that ME/CFS patients have higher B-cells as compared to healthy control and this has been confirmed in various studies ( [68, 69]). In addition to the high cell number, a later study showed that a subset of B-cells in ME/CFS patients have impaired cell signalling function as indicated by lower expression of transient receptor potential melastatin-3 (TRPM3) cation channels on CD19^+^ B-cells in 17 ME/CFS patients, as compared to 19 healthy controls [68, 70]. TRPM3 is an important cell function regulator and the low expression in B-cells implies B-cell function homeostasis is critical to the development of ME/CFS.

Although the changes in T and B-cells population are consistently observed in ME/CFS patients, differences in CD56^Dim^CD16^Bright^ Natural Killer (NK) cells between ME/CFS patients and healthy controls remain inconclusive; for example, a study reported lower CD56^Dim^CD16^Bright^ NK cells for ME/CFS patients [71], while a 2019 study reported no significant differences in NK cell population in 251 ME/CFS patients, compared to 107 healthy controls [65].

Cytokine levels in ME/CFS patients, compared to healthy controls, have also been inconsistent [72, 73]. Cytokines are small proteins involved in cell signalling and communication between humoral and cellular immune response. Pro-inflammatory cytokines such as IL-1, type 1 IFN, and TNF-α generate inflammatory responses. Conversely, IL4, IL10 and transforming growth factor beta (TGF-β) are anti-inflammatory cytokines that reduce inflammation and supress the synthesis of pro-inflammatory cytokines. These cytokines are measured in distinct disorders to evaluate the type of immune response, as well as sources of cytokine production.

A summary of these changes has been illustrated in Tables 1 and 2. However, these findings on immunological abnormalities are controversial as some studies indicated increased inflammation, and infection, associated with immune responses, while others reported decreases for the same population (see Table 3).

**Table 2:** Abnormalities in cellular immune response in ME/CFS

Cellular Cytotoxicity	Modification	References
NK cells (CD16/CD56)	Reduced	[74–76]
T cells, NK cell (DC69)	Reduced	[71, 77, 78]
CD26	Increased	[79]
CD38	Activation	[69, 79]
CD11b, CD11c, CD54, NK cells	Increased	[69]
Perforin	Increased	[71]
Granzyme A, granzyme K (NK cell)	Decreased	[66, 71, 79]
CD8 + T cell	Increased	[71]
VPACR2	Increased	[71]
FoxP3 (T regulatory cells)	Increased	[71]
Perforin	Decreased	[79, 80]
(DDP4)/CD26	Increased	[68]
(DDP4)/CD26	Decreased	[81]
(KAR)NKp46 and CD69	Increased	[82]
CD25 (NK cells)	Decreased	[82]
KIR3DS1 (NK Cells)	Increased	[83]
KIRDL1 (NK cells)	Increased	[83]
CD20, CD21, CD19, CD5 (B cells)	Increased	[68, 69]
CD24 and CD21 + CD38 (B cell)	Increased	[84]
Treg	Increased	[66, 82]
CD8 + memory cell	Increased	[65, 66]
CD4 + T cell	Increased	[66]
CCR6 + Th17	Increased	[66]
Th1/Th2	Increased	[66]
β-NGF	Increased	[85]
CD56Bright NK cell	Decreased	[71]
CD20 + CD5 + B cells	Increased	[86]
(CD25 + /FOXP3 +) CD4 + T cells	Increased	[82]
CD4 + CD25 + T cells	Increased	[71]
Th1 shift to th2	Increased	[64]
TRPM3 (NK cells and B cells)	Decreased	[70]
CD8 + MAIT	Increased	[65]
CD8 + MAIT	Decreased	[66]
CD8 + T cells	Decreased	[66, 71, 82, 87]

**Table 3:** Abnormalities in cytokine response in ME/CFS

Cytokines	Modification	References
IL-8	Increased	[73]
IL-8	Decreased	[72]
IL-5	Decreased	[72, 73]
IL-5	Increased	[73, 88]
IL-12p40	Increased	[89]
IL23	Decreased	[73]
CD40L	Decreased	[72]
IFN-γ	Increased	[71, 72, 88]
IFN-γ	Decreased	[66]
IL16	Decreased	[87, 90]
IL17	Decreased	[66, 72, 87, 90–92]
IL-17f	Increased	[88]
VEGF-A	Decreased	[90]
TNF-α	Increased	[71, 79, 85, 88, 93]
TNF-α	Decreased	[71, 92]
TNF-β	Decreased	[72]
IL-1	Increased	[79, 93]
Il-6	Increased	[73, 79]
IL-1β	Decreased	[72, 92]
IL-1β	Increased	[73]
IL-1a	Increased	[73]
IL-1a	Decreased	[72]
IL-4	Decreased	[92]
IL-4	Increased	[88]
IL-6	Decreased	[92]
IL-7	Increased	[85, 88]
IL-12p70	Increased	[88]
CCL11	Increased	[72]
CXCL10	Increased	[72]
CSF-1	Increased	[89]
IL-10	Increased	[94]
IL-10	Decreased	[71, 72, 92]
CCL11 (Eotaxin-1)	Increased	[85, 88]
CXCL1 (GROα)	Increased	[88]
CXCL10 (IP-10)	Increased	[85, 88]
IL-13	Increased	[88]
M-CSF, GM-CSF, LIF, NGF, SCF	Increased	[88]
CXCL9 (MIG)	Decreased	[88]
TGF-β	Increased	[87, 88]
TGF-ß3	Decreased	[92]
TGF-ß2	Decreased	[92]
TGF-ß1	Decreased	[85, 92]

### Autoimmunity

In addition to immune dysfunction, other evidence has stimulated interest in describing ME/CFS patients developing autoimmune symptoms. The autoimmune symptoms observed in ME/CFS patients involves self-recognising antibodies, known as autoantibodies, are usually IgM and their targets are against neurotransmitter receptors, nuclear and membrane structures [95]. For example, a study showed that 53% of 60 ME/CFS patients were tested for autoantibody against neurotransmitter receptor, muscarinic cholinergic receptor 1 and that these positive patients had a higher mean score of “muscle weakness”, painful node” and “forgetfulness” as compared to healthy control [96]. It is noteworthy that ME/CFS patients with autoimmune symptoms often have comorbidity with other autoimmune diseases such as FM, Hashimoto’s thyroiditis and postural orthostatic tachycardia syndrome (POTS) [93, 97–99].

There are a few suggested triggers for patients with ME/CFS to develop autoimmune symptoms. The first and perhaps most suspected trigger factor is the persistent and recurring virus infections such as EBV, Human Herpes Virus (HHV) and Parvovirus B19 [100]. In 1984, the very first serologic evidence of active infection in ME/CFS was the detection of EBV-caused mononucleosis in 14 patients with chronic symptoms of disabling fatigue [101]. This was further supported by additional studies showing elevated autoantibody levels against EBV nuclear and membrane structures, as well as autoantibodies against neurotransmitters and their receptors [95], increased IgM response against high levels of oxygen reactive species (ROS) and nitric oxide (NO), or the highly reactive oxidant peroxynitrite, respectively [93].

Another trigger is the genetic predisposition type of human leukocyte antigen (HLA) alleles [102]. There are multiple studies on HLA alleles in ME/CFS but most, if not all, of these studies are carried out on 50 or less patients. This has impacted the detection limit to only strong associations between HLA mutation and ME/CFS and at times not reproducible. However, there is one recent study in 2020 by Lande et al., who applied a high-resolution typing on a cohort of 426 patients, and they identified two HLA alleles HLA-C*07:04 and HLA-DQB1HLA*03:03 to have a strong association with the occurrence of ME/CFS [102]. In addition to alleles, there are also studies looking at single nucleotide polymorphism (SNP) in receptors, enzymes or transcription factors with other autoimmune diseases [103].

The third trigger to the development of autoantibody can be the resultant damage mediated by oxidative or nitrosative damage. One study observed higher oxidative stress induced by exercise. They investigated changes in various markers of oxidative stress in 11 CFS patients when compared to healthy controls. They have argued that PEM and muscle pain reported by ME/CFS patients, are in association with identified oxidative stress [104].

Given the clinical heterogeneity of ME/CFS patients regarding the severity, pathomechanisms, co-existing conditions, level of the immune response, etc., unless there are established diagnostic markers to select unified cohort, this data cannot be generalised to all ME/CFS patients [95].

### Mitochondrial dysfunction

Symptoms of ME/CFS such as PEM and fatigue less aided by rest have prompted the investigation of mitochondria and cellular energy metabolism since a deficiency here could contribute to these two key symptoms. Initial studies of mitochondrial function were inconclusive. Assessments of steady-state ATP levels in cells from ME/CFS patients showed conflicting results: decreases were seen in neutrophils, peripheral blood mononuclear cells (PBMCs) and primary skeletal muscle cell cultures [52, 105, 106], while elevated ATP levels were also observed in PBMCs, albeit attributed to nonmitochondrial ATP production [107]. A limitation of these measurements is that steady-state ATP concentration do not solely reflect the rate of mitochondrial ATP synthesis since it is also a product of ATP production by non-mitochondrial pathways and of ATP consumption. More recent studies have instead utilised live-cell respirometry (a real-time, rate-based measure), biofluid metabolomics and other ‘omic technologies. In combination, these techniques can provide detailed insights into the mitochondrial function and also related energy- or substrate-providing pathways such as glycolysis. Not unexpectedly, due to differences in investigative techniques, clinical diagnostic criteria and sample types, there are some differences in findings between studies, yet the overall conclusions appear to be converging as the number of reports grows.

Metabolomics studies in the ME/CFS field have investigated metabolite levels using mass spectrometry (MS) or nuclear magnetic resonance (NMR) spectroscopy. These studies suggest a disturbance in the supply of glucose-derived substrate into the TCA cycle. NMR studies with ME/CFS blood plasma identified decreases in glutamine and ornithine concentrations, suggesting abnormal amino acid metabolism and urea cycle dysregulation [108]. Subsequent work undertaken by the same authors suggested that impaired glycolytic formation of pyruvate may lead to the reduced provision of oxidised substrate for the tricarboxylic acid (TCA) cycle [109]. Others using MS to investigate ME/CFS serum suggested that a deficiency in pyruvate dehydrogenase (PDH) function may instead form a bottleneck for the provision of TCA cycle substrate downstream of glycolysis, PDH being the enzyme that converts pyruvate into the TCA cycle intermediate acetyl-CoA [110]. Whilst the location of the substrate supply defect differed between the studies both suggest TCA cycle disturbance in ME/CFS driven by a reduced supply of glucose-derived substrate. Since these theories are based largely on biofluid measurements, it would be valuable to test them directly in cellular models.

Initial respirometry studies of quiescent (unactivated) or chemically activated ME/CFS patient immune cells found mitochondrial respiration rates compared with healthy controls to be broadly reduced [111] or unchanged [112]. However, subsequent studies of metabolically active lymphoblastoid cell lines from ME/CFS patients using respirometry identified inefficient ATP synthesis by Complex V, the elevation of the proton leak, the maximal respiration rate and its major component (Complex I activity) [113]. These abnormalities were found to correlate with symptom severity and accurately distinguished ME/CFS from healthy control samples [113, 114]. No deficiency in ATP levels nor in the absolute rate of ATP synthesis was observed but an elevation in the activity of the mammalian target of rapamycin complex 1 (mTORC1) was present and could account for the compensatory increase in maximal respiration rates. In agreement with this, whole-cell proteomics of these cell lines revealed an elevated expression of electron transport chain complexes and also in enzymes involved in the TCA cycle, fatty acid and amino acid catabolism (but not those of glycolysis).

The elevated proton leak, respiratory spare capacity and mTORC1 activity were later also observed by others using respirometry and proteomics investigation of PBMCs [115]. Furthermore, the elevated expression of electron transport chain complex subunits (prominently Complexes I and V) and proteins in substrate-providing pathways (such as the TCA cycle) were also reported in PBMC proteomes [116]. Importantly, the upregulation of mitochondrial amino acid and fatty acid catabolism pathways accompanied by a lack of similar glycolytic upregulation in lymphoblastoid cells from ME/CFS patients concurs with the outcomes of the metabolomic studies, suggesting that fatty acids and amino acids are likely to be utilised more readily than carbohydrates by ME/CFS cells. This is in agreement with reports of impaired stimulation of glucose uptake in ME/CFS skeletal muscle cells [117]. Taken together, these reports appear to indicate a dysregulated steady-state involving both deficiency and compensation in ME/CFS cellular energy metabolism.

If these metabolic and mitochondrial pathways are indeed dysregulated as it presently appears, ME/CFS cells could be less able to respond to additional ATP demands (such as those imposed by exertion) since the responding pathways are already activated. This could reflect a cellular equivalent of key clinical features of ME/CFS. If present in cell types or bodily tissues other than immune cells, it may contribute to the unexplained fatigue and PEM which characterise this disease. This is an important area for future exploration.

### Neuroinflammation

As neuroinflammation has been suggested to be responsible for other fatigue-related neurologic diseases, such as multiple sclerosis [118], neuroinflammation has also been hypothesized to contribute to ME/CFS. In a landmark study led by Naktomi et al*.,* the authors revealed activation of microglia and astrocytes in the cingulate cortex (CC), hippocampus (HIP), amygdala (AMY), thalamus (THA), midbrain (MID), and pons (PON) using positron emission tomography (PET) scans. The degree of activation in microglia and astrocytes was also found to be associated with the severity of the patient neuropsychological symptoms [119]. Furthermore, high levels of proinflammatory cytokines (IFN-γ, IL-6, IL-1β, TNF-α) in the peripheral blood and cerebral spinal fluid have also been detected in ME/CFS patients than in healthy controls [120]. Interestingly, an investigation of 44 ME/CFS patients revealed lower levels of granulocyte–macrophage colony-stimulating factor, and higher levels of IL-8 and IL-10 in their spinal fluid. This indicates that there is the immune system in the brain which is resolving an inflammation by secreting anti-inflammatory IL-8 and IL-10 in these patients [121].

Neuroinflammation has been suggested to be triggered or caused by stress, neuronal injuries and infections [122, 123].

### KP, a potential mechanism contributing to ME/CFS pathology and symptoms

Despite the extensive studies and technological advancements, the specific treatment target(s) and diagnosis markers for ME/CFS remains elusive [124]. At present, patients with ME/CFS are identified after a series of disease possibilities have been eliminated and a thorough investigation into their medical history. This thus highlights the need to explore new mechanism(s) involved in the pathology of ME/CFS. Considering that neuroinflammation is a key characteristic of ME/CFS, KP has been proposed as one of the mechanisms that potentiate the progression of ME/CFS. Inflammation usually elevates KP and its activity has been shown to mediate immune suppression, oxidative stress and most importantly NAD + starvation. As confirmed by previous studies, TRP intake has shown significant improvement in anxiety and positive mood in healthy individuals [125]. Further strengthening the notion that KP plays a key role in the pathology of ME/CFS is the current use of supplements such as L-TRP and NADH in ME/CFS patients. TRP is the substrate for this pathway while NADH is the final product of the pathway.

## KP

The KP is the major pathway of TRP metabolism that catabolizes TRP to produce the essential cellular energy source, NAD + . This pathway also produces various metabolites with different effects. Among the KP metabolites, quinolinic acid (QA), a precursor of NAD + , has been shown to have the most neurotoxic properties. It functions as a selective agonist of N-methyl-D-aspartate (NMDA) receptor, and binding to this receptor induces damage to neurons and astrocytes by a massive influx of calcium ions into them. In a neuroinflammation-KP model proposed by Morris et al*.,* in 2016, TRP is actively taken by activated microglia in a chronic inflammatory condition. As a result, TRP increases in the brain after being produced peripherally and passing blood–brain barrier (BBB). Microglia produces QA, then following saturation of KMO enzyme in microglia production of kynurenic acid (KYNA) in astrocytes is promoted [126]. KYNA as a NMDA receptor antagonist competing with QA, is generally considered to be neuroprotective. In addition, it acts as an inhibitor of ionotropic glutamate receptors, a negative allosteric modulator at the α7-nicotinic receptor, an agonist at an orphan G-protein-coupled receptor (GPR35) [127], an agonist of aryl hydrocarbon receptor (AhR) which ultimately leads to a suppression of several inflammatory pathways [128].

**Fig. 1:**
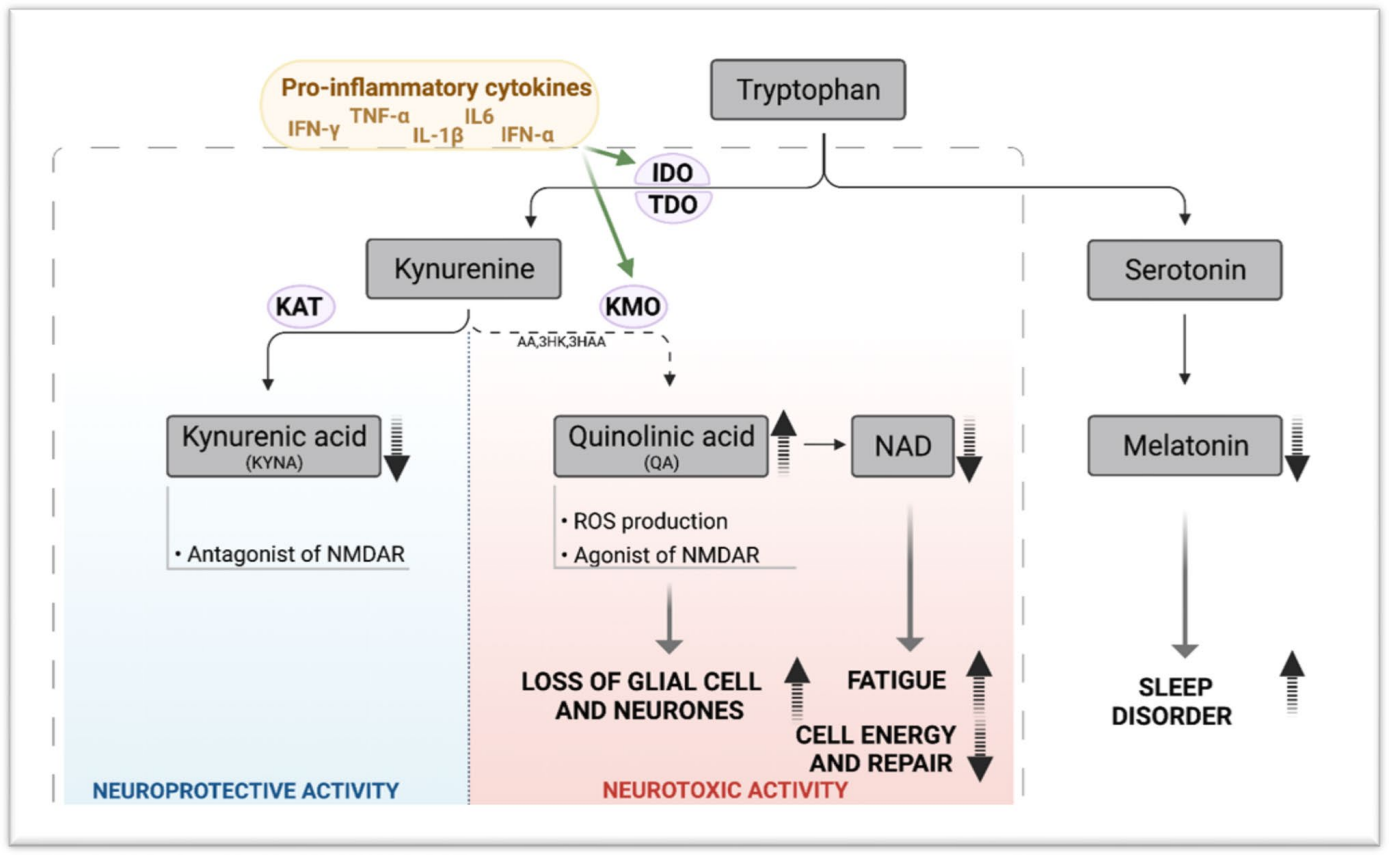
Tryptophan metabolism and KP in inflammatory condition. Created with Biorender.com

In a normal brain, TRP metabolism mediates crosstalk between blood, brain, and the immune system. Metabolism of TRP is tightly balanced between the activity of serotonin (5-HT) pathway and the KP. However, in the presence of pro-inflammatory cytokines, TRP metabolism is skewed towards KP and is activated to produce several neuroactive metabolites including the neuroprotective molecules such as KYNA and the neurotoxin QA instead of 5-HT and melatonin. QA has been proven to be involved in symptoms such as depression and suicidal behaviour in patients [129]. Given that depression has been reported in ME/CFS, QA can be studied as an effective excitotoxin contributing to neuroinflammation in these patients [130]. In addition to QA, NAD + as an important co-factor involved in cellular energy production shows some changes [131]. At first, by increasing QA levels under inflammatory condition, NAD + levels increase, however after saturation of converting enzyme and increase in oxidative activity induced by QA, there is a drop in NAD + production. Decreased levels of NAD + contribute to changes in several functions in cells, as NAD is involved in various cellular processes (e.g., calcium homeostasis, apoptosis, ageing, DNA repair, immunogenicity and transcriptional regulation) [132]. In addition, oxidative stress caused by increase in QA, can activate poly (ADP-ribose) polymerase (PARP), which repairs damage to DNA after oxidative stress. However, its overactivation leads to depletion of NAD + and ATP, consequently disturbance in energy production and mitochondrial function, which have been reported in ME/CFS as other pathologic conditions [133]. TRP metabolism and KP activity under inflammation have been demonstrated in Fig. 1.

### Potential involvement of the KP in ME/CFS

**Fig. 2:**
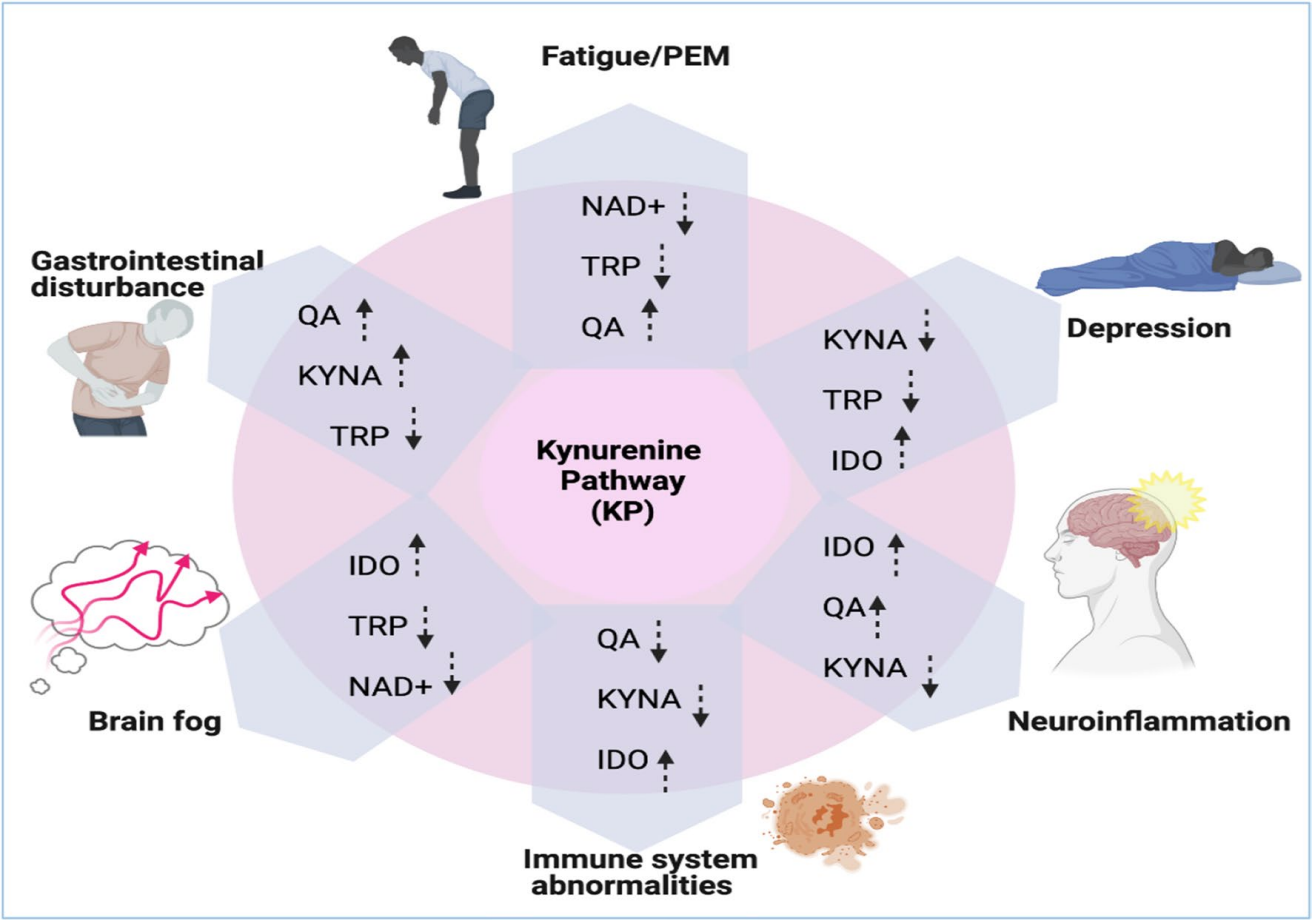
Potential changes in KP contributing to different symptoms in ME/CFS. Created with Biorender.com

In an inflammatory environment, KP can contribute to many pathologic conditions including neuroinflammation, immune system abnormalities, depression, gut microbiota disturbance and central fatigue. These conditions have been reported in ME/CFS patients generating the main symptoms. Therefore, the KP might be the explanatory model linking all the symptoms in ME/CFS. This potential model has been proposed in Fig. 2.

### KP and Fatigue

The KP can be involved in the genesis of central fatigue via different metabolites linked to the pathway. Fatigue can stem from the changes occurred in the level of TRP itself, or its metabolites, or 5-HT production, and other substances linked to TRP levels including dopamine and noradrenalin. In 2012 Anderson et al*.,* suggested that imbalanced TRP metabolism and drop in TRP level led to physico-somatic symptoms such as fatigue, autonomic symptoms, somatic presentation, and hyperalgesia [134]. In addition, the availability of TRP in the brain has been investigated in fatigue. As a result, there is an accumulating body of data focusing on the role of the large neutral amino acid transporter-1 (LAT-1) located on BBB. As TRP must compete with other amino acids such as tyrosine and branched amino acids, therefore, some studies proposed the effectiveness of using branched-chain amino acids as supplements in controlling the amount of TRP in the brain. However, there are downsides to this strategy, such as emerging psychiatric or metabolic symptoms. TRP metabolites are linked to the production of proinflammatory cytokines (PIC)s in the reduction of dopamine levels and elevation of glutamate, which lead to the generation of mental fatigue [126]. In addition, elevated glutamate causes mitochondria dysfunction and lower energy, which can be intensified by both oxidative stress and PICs in neuroinflammatory condition [126]. Some other studies emphasized the importance of 5-HT as the cause of central fatigue. Increased synthesis of 5-HT was reported regarding fatigue after exercise. In addition, there is evidence demonstrating that during the development of exercise, levels of TRP hydroxylase and 5-HT elevates [135]. Similarly, some rodent studies have discussed the role of 5-HT elevation related to the increased level of TRP in CNS [136]. Moreover, some research has emphasized the role of non-esterified fatty acids (NEFAs) in promoting the dissociation of albumin-bound TRP, therefore, increasing free TRP and synthesis of more 5-HT [137]. To investigate the role of 5-HT in CFS, acute TRP depletion (ATD) was performed in a female group of ME/CFS patients. However, there was no significant improvement in symptoms including fatigue, depression, and lack of concentration in these patients. This result highlights the importance of other TRP metabolites rather than 5-HT, or TRP solely [138]. Therefore, focus of other studies in ME/CFS patients was shifted on the importance of KP and elevated KYNA production as the cause of fatigue to be further discussed in “KP in ME/CFS” section.

#### KP in gut microbiota and gastrointestinal (GI) disturbances

Gut microbiota alterations and GI disturbances are very common symptoms in ME/CFS patients. The gut microbiomes of patients with ME/CFS have reported to be less diverse by sequencing bacterial markers when compared to healthy controls. In addition, specific bacterial species often described as pro-inflammatory species were increased, while species frequently referred to as anti-inflammatory were reduced. This result suggested the possible role of microbiota in emerging inflammatory symptoms [139]. In another study, the correlation of these changes was investigated to metabolites in urine and serum of ME/CFS patients, indicating increased production of short-chain fatty acids (SCFA) by microbial fermentation in the gut leading to a deterioration of energy metabolism in these patients [140]. Similarly, a reduction in microbial diversity has been reported in 2021 in ME/CFS patients in both faecal and oral microbiota [141]. These changes in microbiota combination have been linked to TRP metabolism, with subsequent effects on brain function [142]. In 2017, Morris G et al*.,* reported the role of the composition of the microbiota in the level of circulating TRP. This can be due to affecting the KP, or consumption of TRP as an energy source, or to produce 5-HT, or even producing more TRP in the gut. However, the main activity by which microbiota affects the TRP metabolism is through alteration in KP in favour of increasing QA and KYNA [143]. The same relationship was reported between other GI disturbances such as irritable bowel syndrome (IBS) and TRP metabolism. For example, out of 13 serum KP metabolites which were investigated in IBS by liquid chromatography/mass spectrometry (LC/MS), indole-3-lactic acid levels were lowered. However, there is no difference in kynurenine and KYNA levels. Ten out of 13 metabolites showed a significant decrease over the night [144]. The kynurenine and KYNA were associated with adrenocorticotropic hormone (ACTH) (positively) and cortisol/ACTH (negatively) [144]. Similarly, lower indole-3-lactic acid levels were reported in 50 ME/CFS patients versus 50 healthy controls. Notably, half of ME/CFS patients had reported IBS [145]. In addition, Fitzgerald et al*.* reported high levels of plasma kynurenic/TRP ratio in severe IBS cases [146]. More importantly, through the manipulation of TRP metabolites and Indoleamine 2,3-dioxygenase (IDO) production, the microbiota can affect immune response by affecting cell differentiation and engaging AhR [147]. Moreover, changes in TRP metabolisms itself can affect the immune response, which is discussed below.

#### KP and immune imbalance

Overactivation of KP has been proposed as the explanatory model for an immunological connection between malnutrition and infection by Blankfeild (2013). Immune activation occurs particularly by persistent infections involving pathogens including *Borrelia burgdorferi*, human immunodeficiency virus (HIV), mycoplasma and EBV, or elevated commensal Lipopolysaccharides (LPS) in the systemic circulation. As a result, microglia and astrocytes are activated to produce pro-inflammatory cytokines, ROS/Reactive nitrogen species (RNS), Prostaglandin E2 (PGE2), cyclooxygenase-2 (COX-2), as well as other neurotoxins and inflammogens. This neuroinflammation generated by these glial cells, regulates the activation of the KP by changing the transcription and activity of IDO. Other neurotransmitters levels are affected in this condition as well, such as glutamate and dopamine, which are investigated as the source of fatigue in patients [148].

In addition, The KP is involved in generating an immunosuppressive response. In particular, the activation of the KP induces apoptosis in T-cells, inhibition of NK cell function as well as increased differentiation of regulatory T cells [126]. Moreover, most KP metabolites are reported to promote immunosuppression and have anti-inflammatory properties. Kynurenine and KYNA promote anti-inflammatory activity through the activation of hydrocarbon receptors. Activated AhR on antigen-presenting cells and naive CD4 T lymphocytes as well as increased levels of TGF-1 leads to suppression of the differentiation of Th17 and Th1 T cells as another immunosuppressive aspect [149]. In addition, KYNA engagement of this receptor may be a source of IL-6. It should be stressed that the same mechanisms can be activated by persistent pathogen infections or chronically increased intestinal permeability [126].

#### KP and depression/suicidality

Different metabolites of KP have altered in depression. TRP has been reported as significantly lowered in depression while IDO level was not changed [150]. On the contrary, other studies highlighted the association between increased IDO and symptoms of depression. In one study [151], lower TRP levels and higher KYN/TRP ratio in depressed patients with melancholic features were reported. Interestingly, women with severe paranoid personality disorder (PPD) were shown to have their behaviour linked to inflammation and elevated KP and production of inflammatory cytokines. This promptly suggests KP and inflammatory cytokines as potential targets for the treatment [152]. Similar observations have been reported in suicidal patients. [153, 154]. A study investigating the KP in patients with depression reported KYNA/QA ratio was lower in patients with major depression disorder as compared to healthy control. There was no significant difference in concentration of C-reactive protein and inflammatory cytokines between the patient and healthy control cohort. This suggested an imbalance in KYNA and QA in the condition of depression [155]. The importance of KYNA was further highlighted in another study that reported a lower mean KYNA concentration in 58 patients with major depression as compared to 189 normal [156]. In addition, the importance of elevated QA was confirmed by investigating CSF samples of 64 medication-free suicidal patients and 36 controls, by using gas chromatography–mass spectrometry and high-performance liquid chromatography. They found that QA, but not KYNA, was significantly higher in the CSF of suicide attempters (P < 0.001) [157]. On the other hand, lower levels of another neuroprotective KP metabolite (picolinic acid (PIC)) has been reviewed in suicidal attempts [158]. Therefore, collective data suggest that KP may contribute to depression and suicidality, which is one of the symptoms reported by ME/CFS patients.

#### KP in neurogenerative and cognitive dysfunction

There is no study investigating the role of KP in generating brain fog and cognitive impairment in ME/CFS. However, other studies have investigated the importance of KP in neurodegeneration and cognitive impairments in other diseases. For example, in schizophrenia QA has been studied as the neurotoxin which leads to cognitive deficits [159]. In addition, the role of KYNA has been highlighted in several studies in generating learning and memory abnormalities, and cognitive flexibility in these patients [160, 161]. Another disease in which changes in TRP metabolites have been reported is Alzheimer’s disease (AD), indicating the involvement of this pathway in the pathology of the disease. In AD, inhibitors of the KYN pathway were neuroprotective in AD mouse models (79,80), similarly in fly and worm models of AD and Parkinson’s disease [162]. In addition, a lower concentration of TRP and increased Kyn/Trp indicating degradation of TRP was reported in AD patients in 2020 [163]. Another recent research in 2021 has reported lower metabolite concentrations of TRP pathway metabolites in both urine (*n* = 560) and serum (*n* = 354) samples of AD patients [164]. This data can emphasize the role of TRP metabolism in neurodegeneration and central symptoms. Altogether, evidence regarding the involvement of KP in neurodegeneration and cognitive dysfunction can highlight the possible role of KP in generating neurological symptoms of ME/CFS, which should be investigated in the future.

#### KP in ME/CFS

Although there are some studies connecting various symptoms mentioned above with KP, studies that have investigated KP in ME/CFS are limited. The potential involvement of the KP in the pathogenesis of ME/CFS patients was highlighted by a 2003 study that reported significantly higher concentration of free TRP in ME/CFS patients versus 11 age- and sex-matched controls [165]. This trend was again found in another study where a higher free TRP level was measured in sera of 23 patients with the ME/CFS as compared to 42 healthy controls [166]. The results from these studies indicated that the KP is likely to be dysregulated in patients with ME/CFS. In 2019, a model named the IDO metabolic trap was suggested for the aetiology of ME/CFS. In this model, the backup enzyme in KP, IDO2, is absent or dysfunctional due to several reasons including genetic mutations. In addition, substrate inhibition of IDO1 combined with disrupted LAT-1 have led to proposing a driving model of KP in ME/CFS patients. However, this model is purely mathematical and demands further testing [167]. Another study conducted in 2019 reported a lower ratio of KYN/TRP and 3-HK in CFS patients when compared to healthy controls [168]. Similarly, another recent study conducted in 2021 has reported changes indicating lower neuroprotective activity of KP in ME/CFS and FM in 49 CFS patients; 57 FM patients; versus 54 healthy controls. The ratio given by KA/QA was lower for CFS patients compared to healthy controls, as well as lower levels of anthranilic acid (AA) and no significant changes in other metabolites [169]. Lastly, degradation of amino acids and dysregulated mitochondrial function have been reported in ME/CFS patients by Fisher et al., in 2020 and 2021, which highlights the importance of KP in relation to mitochondrial dysfunction and energy production in these patients [11, 113]. Finally, a recent study showed that patients with ME/CFS have a higher level of 3HK (*p* = 0.037) while lower levels of KYN (*p* = 0.012) as compared to 40 sex- and age-matched healthy controls. Interestingly, among the ME/CFS patients with the start of the disease, those who had recovered from an infection had lower levels of KYN (p = 0.034) as compared to those who did not have an infection. Furthermore, there were no correlations between the KP, cytokines and fatty acids in the study cohort. This implied that the changes of these biological components are independent of each other while being associated with the pathology of the disease [170].

## Conclusion

Although ME/CFS has been recognised in patients for years, there has not been any improvement in its diagnosis and treatment. ME/CFS is a long-term serious disease that affects not only the body system but also patient’s usual activities. Patients with ME/CFS often display severe fatigue and sleep problems, with a portion of cases confined to bed. The key factor undermining the diagnosis and treatment efficacy of ME/CFS is the failure to identify the cause of this disease. Most, if not all, available treatments are symptom-driven and have only provided relief to the symptoms but not cure the disease. Another challenge faced in treating ME/CFS is the complicated nature of the disease. This has led to a lack of unified diagnostic guideline to identify and treat a patient with ME/CFS [94, 114, 171]. There have been advances in research on several aspects of ME/CFS including triggering infections, immunological aspects, metabolic changes, and genetic disorders of ME/CFS. However, data obtained from these studies were either contradicting or inconclusive. This might be due to the heterogenicity in the study materials from ME/CFS patients, different severity of the disease, prior infections and medication history of the patients. Most importantly, the clinical data and criteria used in this investigation were subjective as it was based on patient questionnaires. Therefore, the importance of exploring a new avenue in the disease has been highlighted and KP has been suggested to be one of the key players that offers a potential impact on the pathology of ME/CFS.

KP has emerged as a potential candidate for the pathology of ME/CFS due to its strong association with neuroinflammation and its bioactive metabolites. Firstly, this pathway is involved in many mechanisms generating the pathologic hallmarks of ME/CFS, including the dysregulated immune response and inflammation. Secondly, it is the metabolites produced along the pathway that could contribute to various symptoms of ME/CFS. For instance, given that low energy level and fatigue are the main features of ME/CFS, KP is the main pathway in production of (NAD +) as the essential cellular energy source. In addition, alterations in various metabolites involved in this pathway have been reported in ME/CFS as discussed above, and these changes are linked to the type of immune response such as secretion of various cytokines and chemokines.

Altogether, the KP may play an important role in the pathology of ME/CFS. Therefore, understanding how the KP profile changes through the course of ME/CFS, particularly with regard to its role in contributing to various hallmarks of the disease will be a research priority. Then, it will be worth considering an integrated functional approach to better understand the complex relationship between the KP and various other metabolic or immunological dysregulated pathways in ME/CFS. This could lead to new promising disease management strategies, including the identification of blood biomarkers for disease severity, progression and response to treatments. It is noteworthy to mention that inhibitors and prodrugs targeting KP enzymes are available and are currently being assessed for other diseases. A number of inhibitors of IDO1 have shown promising results in in vitro and animal studies and are presently in clinical trials for various diseases [172]. Nonetheless, changing the management of patients with ME/CFS will need more research and close collaboration between researchers, clinicians and patients to achieve a significant medical outcome “from bench to the bedside”.

## Abbreviations

3HK: 3-Hydroxykynurenine
5-HT: 5-Hydroxytryptamine
AD: Alzheimer’s disease
AA: Anthranilic acid
AhR: Aryl hydrocarbon receptor
BBB: Blood brain barrier
CFS: Chronic fatigue syndrome
CoQ10: Coenzyme Q10
EBV: Epstein–Barr virus
FM: Fibromyalgia
GI: Gastrointestinal
HLA: Human leukocyte antigen
IgM: Immunoglobulin M
IDO: Indoleamine 2,3-dioxygenase
IFN-γ: Interferon gamma
IL: Interleukin
IBS: Irritable bowel syndrome
KYNA: Kynurenic acid
KP: Kynurenine pathway
LAT-1: Large neutral amino acid transporter-1
LC/MS: Liquid chromatography/mass spectrometry
ME: Myalgic encephalomyelitis
NAM: National academy of medicine
NK Cells: Natural killers cells
NAD +: Nicotinamide adenine dinucleotide
NMDA: *N*-Methyl-d-aspartate
PEM: Post-exertional malaise
QA: Quinolinic acid
SNP: Single polymorphism
SEID: Systemic exertion intolerance disorder
Th: T helper
CCC: The Canadian consensus criteria
TRP: Tryptophan
TNF-α: Tumour necrosis factor alpha
MS: Mass spectrometry
NMR: Nuclear magnetic resonance
mTORC1: Mammalian target of rapamycin complex 1
PBMC: Peripheral blood mononuclear cell
TCA: Tricarboxylic acid
